# Annotations

**Published:** 1900-06-02

**Authors:** 


### Annotations.

### The Penal Powers of the General Medical Council.

Although medical practitioners well know how absolute is the power over them possessed by the General Medical Council in all that concerns professional conduct, we fancy that many little recognise how far its authority goes in reference to other affairs. It is well then to be reminded that the Council has the power to consider, and practically to re-try, any case in which a registered medical practitioner has been convicted of a misdemeanour, and to inflict upon the defendant a punishment which may happen to be quite disproportionate to any that is recognised by the ordinary law for a similar offence. At the meeting of the Council held on May 24th the consideration was resumed of the case of a registered medical practitioner, who had been summoned to appear before the Council on the following charges: "(a) that he was on July 10th, 1899, at the Warwick Borough Sessions convicted of being drunk and disorderly; (b) that he was on November 13th, 1899, at the Warwick Court House, convicted of misdemeanour—namely, of assaulting and beating Ellen Simpson." In the result the President announced that the Council had directed the Registrar to erase the name from the Medical Register. Other cases also were considered—practically tried, seeing that some of the accused were punished, and others let off—the clause under which they were brought before the Council being that in which it is stated that "if any registered medical practitioner shall be convicted . . . of any felony or misdemeanour the General Council may, if they see fit, direct the registrar to erase the name of such medical practitioner from the register." We do not quarrel with the exercise of these powers by the Council; on the contrary, we are sure that one of the most important functions of that body is to keep clean the register. We do feel, however, that the exercise of these judicial functions is a duty involving great responsibility, and that it would be well if some means could be devised by which the personal element could be eliminated from the proceedings. It is not fair that it should be left to the medical men of any district to prosecute their recalcitrant brethren. Local option in medical ethics is a far from satisfactory arrangement, and it would be much better if the maintenance of a uniform standard could be undertaken by some widely-spread organisation than that the initiation of penal proceedings should be left, as at present, to the public spirit or the private spite of individuals.

### The Stock Diseases of Humanity.

Our attention has been drawn to the woes of the dyspeptic by a letter from "An Out-patient," who protests against the very small degree of attention which his case receives at the hands of his doctor. He complains that a curt "How are you?" is followed "in a moment after the reply" by a prescription, that no investigation is made, that no directions as to diet are given; and then he makes a naïve suggestion to the effect that brief instructions as to diet and régime, framed according to the "Stock Diseases of Humanity," might be printed for general use. We like that phrase. There is a practical, retail tradesman-like ring about it. We understand that young ladies can dress both cheaply and smartly at ready-made shops if they do but have the good luck to "run" to "stock sizes." We are not sure that the same thing does not happen to the city clerk, and if the outward form of man can be so nearly estimated in averages as to be fitted by one or other of a moderate number of "stock sizes," why not his liver? Clearly for average man salvation is to be found in the tabulation of the "Stock Diseases of Humanity." We have heard, indeed, of "stock bottles" as things already used in club practice, and by working upon these as a basis, and "fitting" them to "stock diseases," practical medicine might evidently be greatly simplified. Of course, the "fitting" would be a difficulty, and a skilled hand in the shape of a doctor might be wanted now and then, especially for those "odd shapes" whose maladies do not conform to the "Stock Diseases of Humanity." Still, we can quite understand that the man in the street, to whom all great things seem so simple, would be much gratified to find the mystery of medicine brought somewhat in line with what he would call ordinary business principles. Let him be of good heart. Things are not so bad as they seem. Even doctors are not quite beyond hope; and we have no doubt that, if we could but peep into the brains of some of our great consultants, we should find them working on quite ordinary lines, "fitting" their favourite mixtures to their dyspeptic patients much as the "young lady" over the way fits, her customers with ready-made costumes.

### Certificates.

It is difficult to understand the frame of mind of a man who signs a series of blank certificates of "cause of death" and leaves them for his *locum tenens* to use according to his discretion. Yet that such a thing is not infrequently done is shown by the evidence given in a case recently brought before the stipendiary magistrate at Leeds. It seems probable that much of the slackness of professional conscience in regard to this matter which evidently exists is the result of the multitude of printed certificate "forms" which medical men are constantly pestered to sign. These printed forms often contain statements which those who sign them cannot know of their own knowledge. An out-patient who groans and complains of a pain, say, in his back, gets a certificate, saying, "I hereby certify that John So-and-So is ill and unable to work." How does the doctor know? Again, a certificate is wanted for a child going to a convalescent home. On the printed form there are certain questions. "Is the child subject to fits?" "Has he had measles?" "Does he wet his bed?" "Can he dress himself?" and so on; the answers to all of which the doctor can merely take down from what the mother tells him. The odd thing is that he thinks it no harm to sign his name to all this rigmarole, and thus make himself responsible for every statement which it contains. We cannot but think that it is the habit of signing the ridiculous printed forms which are being constantly presented for "club," School Board, and other purposes, that makes doctors so careless, as the case above mentioned shows them sometimes to be, in regard to the use of their signatures. Little errors lead to great ones, and if a medical man—or, indeed, anyone else—once begins to put his name to things beyond his own knowledge, there is no end to the difficulties into which he may be led.

